# Chemotherapy-Induced Alopecia by Docetaxel: Prevalence, Treatment and Prevention

**DOI:** 10.3390/curroncol31090423

**Published:** 2024-09-23

**Authors:** Aleymi M. Perez, Nicole I. Haberland, Mariya Miteva, Tongyu C. Wikramanayake

**Affiliations:** 1 Dr. Phillip Frost Department of Dermatology and Cutaneous Surgery, Miller School of Medicine, University of Miami, Miami, FL 33136, USA; aperez31980@med.lecom.edu (A.M.P.); ni315488@ucf.edu (N.I.H.); mmiteva@med.miami.edu (M.M.); 2 Lake Erie College of Osteopathic Medicine, Bradenton, FL 34211, USA; 3 College of Medicine, University of Central Florida, Orlando, FL 32827, USA; 4 Cancer Control Program, Sylvester Comprehensive Cancer Center, Miller School of Medicine, University of Miami, Miami, FL 33136, USA

**Keywords:** docetaxel, alopecia, prevention, minoxidil, scalp cooling

## Abstract

Docetaxel is a commonly used taxane chemotherapeutic agent in the treatment of a variety of cancers, including breast cancer, ovarian cancer, prostate cancer, non-small cell lung cancer, gastric cancer, and head and neck cancer. Docetaxel exerts its anti-cancer effects through inhibition of the cell cycle and induction of proapoptotic activity. However, docetaxel also impacts rapidly proliferating normal cells in the scalp hair follicles (HFs), rendering the HFs vulnerable to docetaxel-induced cell death and leading to chemotherapy-induced alopecia (CIA). In severe cases, docetaxel causes persistent or permanent CIA (pCIA) when hair does not grow back completely six months after chemotherapy cessation. Hair loss has severe negative impacts on patients’ quality of life and may even compromise their compliance with treatment. This review discusses the notable prevalence of docetaxel-induced CIA and pCIA, as well as their prevention and management. At this moment, scalp cooling is the standard of care to prevent CIA. Treatment options to promote hair regrowth include but are not limited to minoxidil, photobiomodulation (PBMT), and platelet-rich plasma (PRP). In addition, a handful of current clinical trials are exploring additional agents to treat or prevent CIA. Research models of CIA, particularly *ex vivo* human scalp HF organ culture and *in vivo* mouse models with human scalp xenografts, will help expedite the translation of bench findings of CIA prevention and/or amelioration to the clinic.

## 1. Introduction

Key Points:

- Alopecia, or hair loss, is one of the most common adverse effects associated with docetaxel chemotherapy.
- The fear of chemotherapy-induced alopecia can cause up to 14% of patients to consider rejecting the recommended optimal life-saving cancer treatment.

Along with paclitaxel and cabazitaxel, docetaxel is a widely used taxane chemotherapeutic drug. It is a semi-synthetic analog of paclitaxel and differs from paclitaxel at two positions in its chemical structure. Docetaxel is produced by the esterification of a noncytotoxic precursor extracted from the needles of the European yew, *Taxus baccata* [1]. As an anti-neoplastic, cytotoxic drug, it works primarily as a microtubule-stabilizing agent that promotes microtubule assembly and polymerization, inhibiting the cell cycle in the G2/M phase [2]. It was approved by the US Food and Drug Administration (FDA) in 1996 to treat metastatic breast cancer [2]. Additionally, docetaxel shows strong pro-apoptotic activity and antiangiogenic effects, both *in vitro* and *in vivo* [3,4]. With its multiple anti-cancer mechanisms, docetaxel has been approved by the FDA for the treatment of breast cancer, ovarian cancer, prostate cancer, non-small cell lung cancer, gastric cancer, and head and neck cancer [2,3,4], However, docetaxel can cause many adverse effects [2,3,5]. Alopecia, or hair loss, is one of the most common adverse effects associated with docetaxel chemotherapy [2,6].

Chemotherapy-induced alopecia (CIA) refers to partial or complete hair loss due to chemotherapy, occurring most notably on the scalp, but it can also affect the eyebrows, eyelashes, and beard [7,8]. Hair follicles (HFs) undergo cycles of growth (anagen), regression (catagen), and relative quiescence (telogen). At any given time, approximately 80–90% of scalp HFs are in the anagen phase when the matrix cells in the HF rapidly proliferate [9], making them susceptible to docetaxel-induced cell death [10]. Once the proliferation is abruptly inhibited, the hair shaft sheds or breaks, resulting in alopecia [11]. CIA can be observed as soon as 4 weeks after the initial chemotherapy treatment, and hair often grows back within 6 months of chemotherapy cessation [12].

Aside from the physical aspect of alopecia, CIA also exerts a negative impact on patients’ quality of life. Although some patients might find CIA to be the least burdensome side effect [5], others classify it as a traumatic experience even though it is considered an adverse effect that usually does not need medical attention [13]. Hair loss makes patients feel visibly ill and makes it difficult to keep their cancer status private [14,15]. In addition, patients feel as if they have lost their personal identity and are now labeled by society as “cancer patients” [15]. Patients suffering from CIA also experience negative body image and consequent loss of self-confidence [13,15]. The fear of CIA can cause up to 14% of patients to consider rejecting the recommended optimal life-saving cancer treatment [8].

CIA is usually reversible; however, there is increased evidence that persistent, or permanent, chemotherapy-induced alopecia (pCIA) can occur. pCIA is defined as incomplete or no hair regrowth after 6 months of chemotherapy cessation [16,17]. pCIA has been associated with the use of taxanes, busulfan, cyclophosphamide, anthracyclines, carboplatin, tamoxifen, and alfa-2a interferon [18,19,20]. pCIA can have significant psychological consequences, such as having a lower body image score with an increased reluctance to go out in public [17,21]. Patients reflecting on their pCIA call it “devastating”, “depressing”, and “very upsetting” [17]. Therefore, it is paramount to prevent CIA to decrease patients’ anxiety and distress, which could also improve compliance with cancer therapy [8].

## 2. Prevalence of Docetaxel-Induced CIA

Key Points:

- Increased alopecia prevalence is associated with docetaxel dose of >55 mg/m^2^.
- Increased alopecia prevalence is associated with docetaxel combination therapies.

Docetaxel causes CIA at a high prevalence when the dosage reaches 55 mg/m^2^ [10]. The basic recommended dosing consists of administering docetaxel intravenously at 75 mg/m^2^ every 3 weeks, for 3–10 cycles depending on the cancer [3]. When docetaxel is given as monotherapy at 75 mg/m^2^, a prevalence of alopecia was seen in 34.3–42.9% of patients [6,22,23] (Table 1), comparable with that of paclitaxel [24]. However, a significantly higher prevalence of 83.3% was reported with a dose of 100 mg/m^2^ (Table 1) [25]. These observations suggest a possible dose-dependent correlation between docetaxel and the occurrence of alopecia [10].

In addition to monotherapy, docetaxel is also used in combination therapy to treat advanced cancers such as metastatic breast cancer, metastatic non-small cell lung cancer, and advanced gastric and squamous cell/head and neck cancer [3]. Although docetaxel is used in various cancer treatments, half of the CIA studies come from breast cancer therapies. Whereas the varied methodologies and stratifications have made it difficult to compare the results from the limited number of studies on docetaxel-induced alopecia, there is a trend of increased alopecia prevalence in docetaxel combination therapies. The CIA prevalence rates of 74–90% with 60 mg/m^2^ of docetaxel in combination therapy and 44–100% with 75 mg/m^2^ or 85 mg/m^2^ of docetaxel in combination therapy are significantly higher than the 34.3–42.9% prevalence for docetaxel monotherapy mentioned above [12,26,27,28,29,30,31,32] (Table 1). The variations in rates are likely due to docetaxel being combined with different agents, some of which—such as capecitabine—have also been associated with alopecia [10].

**Table 1:** Prevalence of alopecia upon docetaxel treatment. All treatments were every 3 weeks unless specified.

Docetaxel Dose	Docetaxel Regimen	Cancer Type	Adjunctive Therapy	Prevalence	References
**Monotherapy**					
**75 mg/m^2^**	10 cycles ^	Metastatic castration-resistant prostate cancer, n = 379 men, Mean age = 68	-	34.3%	[6]
	4 cycles	Non-small cell lung cancer, n = 288, Median age = 57	-	37.7%	[22]
	Not included	Metastatic castration-resistant prostate cancer, n = 49, Median age = 68.5	-	42.9%	[23]
**100 mg/ m^2^**	6 cycles	Recurrent breast cancer, n = 6, Mean age = 55	-	83.3%	[25]
**Combination Therapy**					
**40 mg/m^2^** every 2 weeks *	12 cycles ^	Metastatic castration-naïve prostate cancer, n = 35, Median age = 68	Androgen deprivation therapy	74%	[26]
**60 mg/m^2^**	3 cycles	Locally advanced gastric cancer, n = 20 Mean age = 58.1	Bevacizumab 7.5 mg/kg Cisplatin 60 mg/m^2^ Capecitabine 937.5 mg/m^2^	90%	[28]
**75 mg/m^2^**	6 cycles ^	HER-2 positive metastatic breast cancer, n = 276, Mean age = 55.6	Trastuzumab 600 mg ^@^ Pertuzumab 420 mg/kg	46.8%	[29]
	6 cycles ^	Non-small cell lung cancer, n = 9, Mean age = 67	GSK3052230 ^#^	44%	[32]
	4 cycles	Locally advanced or early HER2-negative breast cancer, n = 65, Median age = 52	Capecitabine 1000 mg/m^2^	76.9%	[27]
	4 cycles	Locally advanced or early HER2-negative breast cancer, n = 74, Median age = 52	Epirubicin 75 mg/m^2^	91.4%	[27]
	4 cycles	Breast cancer, n = 9, Mean age = 49	Cyclophosphamide 600 mg/m^2^	100%	[12]
	4 cycles	Early-stage breast cancer, n = 30 Mean age = 53.96		96.7%	[31]
**85 mg/ m^2^**	4 cycles adjunctive, then 4 cycles docetaxel	Locally advanced breast cancer, n = 100, Median age = 47	Doxorubicin 40 mg/m^2^ Cyclophosphamide 600 mg/m^2^ (delivered in PEG-coated liposomes)	64.3%	[30]

^ Median number of cycles; * Dosage is equivalent to 60 mg/m^2^ every 3 weeks; ^@^ Trastuzumab monotherapy showed less than 3% alopecia [33]; ^#^ GSK3052230 is a decoy receptor for FGF ligands, tested for use in FGFR1-amplified non-small cell lung cancer.

## 3. Docetaxel-Induced Persistent or Permanent CIA (pCIA)

Key Points:

- Taxane-based treatment is associated with an eight times greater likelihood of inducing pCIA compared to other chemotherapeutic agents.
- Docetaxel is associated with a higher risk for pCIA than paclitaxel.

Compared to other chemotherapeutic agents, taxane-based treatment is associated with an eight times greater likelihood of inducing persistent or permanent CIA (pCIA) [16,21,34], docetaxel being associated with a higher risk for pCIA than paclitaxel [16,17]. In a retrospective study in the UK, while 14 of 138 (10.1%) patients on paclitaxel reported pCIA, 57 of 245 (23.3%) patients on docetaxel reported pCIA [17]. In addition to inducing massive mitotic defects and apoptosis in the proliferating keratinocytes in the anagen HF matrix, paclitaxel was shown to also cause damage in the HF bulge epithelial stem cells in *ex vivo* human HF organ culture [35]. Damage to the stem cells, including epithelial–mesenchymal transition (EMT), likely decreases the HF’s ability to regenerate, thus leading to persistent alopecia [35]. It is yet unknown whether unwanted EMT also plays a role in docetaxel-induced pCIA.

**Figure 1:**
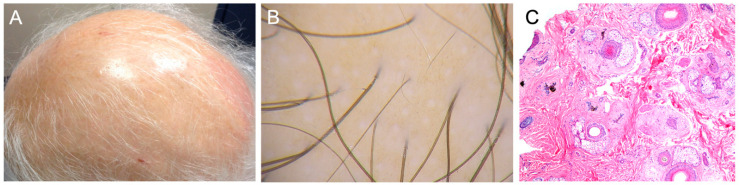
Clinical, trichoscopic, and histologic presentations of persistent/permanent chemotherapy-induced alopecia (pCIA). (**A**) pCIA in an elderly woman demonstrating significantly reduced hair density that is most pronounced on the vertex (crown). (**B**) Trichoscopy of pCIA (different case) reveals significantly reduced hair density with hair shaft variability and short regrowing hairs. Note the irregular pigmented network due to the sun reaching more easily the skin surface devoid of good hair density (FotoFinder, ×40). (**C**) Histology of pCIA shows decreased follicular density with an increased number of telogen follicles (particularly telogen germinal units), which corresponds to anagen arrest. There is follicular miniaturization (Hematoxylin and Eosin, ×10).

Clinically, pCIA is most often described as nonscarring alopecia with diffuse hair thinning. The pattern of hair loss is similar to androgenic alopecia, affecting the temples, vertex, and mid-frontal scalp (Figure 1A) [36,37]. There is a decrease in hair density and a reduction in hair thickness under trichoscopy (Figure 1B) [21,37]. Like androgenic alopecia, there is follicular miniaturization and vellus hair (Figure 1C) [16,38]. The histology of pCIA also shows decreased follicular density with an increased number of telogen follicles (particularly telogen germinal units), which corresponds to anagen arrest (Figure 1C) [16,38].

In addition to scalp alopecia, patients receiving docetaxel reported partial or no regrowth in their eyebrows (53.5%, 5.7%, respectively) and eyelashes (42.5%, 1.8%, respectively) [17]. pCIA can be further categorized by its severity into two grades, assessed at 18 months after chemotherapy cessation: Grade 1 refers to partial alopecia, and Grade 2 refers to complete alopecia needing a wig [16]. A study in Spain following pCIA in adjuvant docetaxel revealed that 35–52% of patients receiving a cumulative dose of >400 mg/m^2^ of docetaxel presented with Grade 1 pCIA, with an additional 10.06% of patients presenting with Grade 2 pCIA; no patients receiving the lower cumulative dose of 300 mg/m^2^ of docetaxel presented with Grade 2 pCIA [16]. These observations provide strong evidence that docetaxel causes pCIA, and the incidence and severity are dose-dependent.

Regarding susceptibility to pCIA, studies have shown that both genetic predisposition and age-related factors can play a role. In a study investigating the predisposing factors of pCIA among women treated with docetaxel-based chemotherapies for breast cancer, 71 (33%) of 215 patients were diagnosed with Grade 2 pCIA while 144 (67%) patients had complete hair regrowth [39]. A single-nucleotide polymorphism (SNP) at rs1202179 was found to be associated with the development of pCIA (odds ratio, 3.79; 95% CI, 2.17–6.62; *p* = 3.05 × 10^−6^) [39]. This variant is linked with the ABCB1 gene, which encodes an efflux pump that normally eliminates docetaxel from cells, likely including the HF stem cells. However, risk allele C is associated with decreased ABCB1 expression, consequently leading to reduced docetaxel elimination and, thus, its intracellular accumulation [39]. The same study also detected a higher prevalence of pCIA in women of older age [39], consistent with findings reported previously in post-menopausal versus peri- or pre-menopausal women [16,17]. Various factors can play a role in alopecia post-menopause, such as medications, chronic disease, telogen effluvium, or female pattern hair loss [16]. It is not known whether ABCB1 expression levels decrease with age, which would also help explain the increased incidence of pCIA with age. Additional extensive research to identify other predisposing factors for pCIA will provide the foundation to develop new strategies to prevent pCIA.

## 4. Treatment for CIA and pCIA

Key Points:

- There is currently no cure for pCIA, and the treatment outcomes are mostly disappointing.
- Various clinical trials are investigating the use of minoxidil, photobiomodulation, or platelet-rich plasma as a treatment for pCIA/CIA.

**Figure 2:**
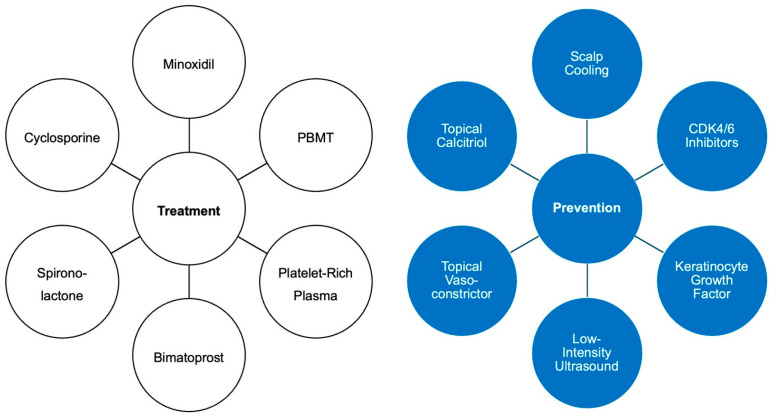
Treatment and preventative approaches for CIA and pCIA.

In CIA treatment, the goal is to achieve complete hair regrowth to a pre-chemotherapy stage. In pCIA treatment, the goal is to improve hair density to a level that allows the patient to not wear a wig or other camouflaging accessories. However, this is often not possible (authors’ own experience). Currently, there are several approaches to promoting hair regrowth in the treatment of CIA (Figure 2).

A commonly used treatment is minoxidil, a vasodilator first used for the treatment of severe refractory hypertension, with adverse effects of hypertrichosis and hair growth [40]. Minoxidil is thought to stimulate hair growth by inducing angiogenesis via activating prostaglandin-endoperoxide synthase 1 and upregulating vascular endothelial growth factor expression. Minoxidil should be initiated after chemotherapy discontinuation due to its anagen prolongation effect [41]. A 2% or 5% topical minoxidil solution or 5% foam has been widely used to treat CIA and even pCIA [14,34,40,41,42,43]. In addition to topical treatment, low-dose oral minoxidil, which is being investigated for various hair loss conditions including pCIA (NCT03831334) and androgenetic alopecia [19,40], may help patients with poor compliance or intolerance to topical minoxidil [19,40], and/or add additional benefits when used concomitantly with the topical formulation.

Photobiomodulation (PBMT), or low-level laser therapy, has also shown efficacy in promoting hair regrowth. While PBMT was initially used for wound healing and musculoskeletal disorders, it has been shown to be effective in treating male and female pattern hair loss and alopecia areata, with further investigation needed on its effectiveness on CIA [14,44,45,46,47]. PBMT promotes hair regrowth by inhibiting inflammation, increasing growth factor expression, ATP production, and blood circulation, which induce the HF to enter the anagen phase [48]. The proposed mechanisms include PBMT acting on the mitochondria, causing photodissociation of inhibitory nitric oxide from cytochrome c oxidase, and altering cell metabolism [49]. When investigating PBMT’s effect on CIA, a study using a neonatal rat model showed that hair regrowth occurred 5 days earlier with PBMT compared to the sham-treated rats [50]. The World Association of Photobiomodulation Therapy (WALT) has prescribed PBMT parameters for prophylactic and therapeutic use in supportive care for cancer therapy, including CIA [51]. Currently, three clinical trials are investigating the efficacy of PBMT in promoting hair regrowth after CIA, with the last clinical trial testing focusing on CIA due to docetaxel administration (NCT04036994, NCT05177289, NCT05397457).

Platelet-rich plasma (PRP) is another treatment for CIA. PRP is extracted from the patient’s blood and is enriched in a variety of growth factors, including vascular endothelial growth factor (VEGF), platelet-derived growth factor (PDGF), and insulin-like growth factor, which act on stem cells of the HF and cause follicular proliferation through the ERK pathway [52,53]. Concerns were raised that the release of growth factors could stimulate angiogenesis close to the PRP application sites in the scalp, which may lead to tumor lymphangiogenesis by VEGF and PDGF and distant metastases [54]. However, an application analysis of 163 sentinel lymph node biopsies in patients with breast cancer demonstrated that PRP showed zero distant metastases and local recurrences as well as a 100% 30-month overall survival [55]. Although no enhanced hair regrowth was observed in rat models with CIA upon PRP treatment [56], a meta-analysis reported evidence—despite being low-quality due to inconsistency and risk of bias—that PRP injections increased hair density in androgenetic alopecia and alopecia areata, raising interest in further research for CIA [57]. Currently, a clinical trial is investigating the effectiveness of PRP therapy for the treatment of CIA and pCIA after cancer therapy in women with breast cancer (NCT04459650) at Memorial Sloan Kettering Cancer Center and is expected to be completed by June 2025 and provide the results of PRP treatment in patients.

Other agents have also shown positive outcomes in treating CIA caused by other chemotherapeutic drugs (Figure 2). These agents include bimatoprost (a synthetic analog of prostaglandin F2α), spironolactone (a synthetic aldosterone receptor antagonist), cyclosporine (an immunosuppressive calcineurin inhibitor), and various antioxidants [14,34,58,59,60,61]. These agents need to be investigated for their efficacy in promoting hair regrowth after docetaxel-induced CIA.

## 5. Preventative Measures

Key Points:

- The successful prevention of CIA is classified as <50% hair loss.
- Scalp cooling administered by healthcare professionals remains the only FDA-cleared measure to prevent CIA by docetaxel.

The scalp cooling system first received FDA clearance in 2015 in the United States as a preventative measure against CIA [14]. There are two proposed mechanisms for preventing hair loss. First, scalp cooling causes vasoconstriction, leading to decreased blood flow and reduced chemotherapy agents in the vicinity of the HFs. Second, cooler temperature reduces cellular metabolism in the follicular cells. [13]. Most studies quantify the severity of CIA through the Dean scale, with successful prevention of CIA defined as <50% hair loss (grade 0–2) [62,63,64,65,66]. When comparing the efficacy of scalp cooling between taxanes, regimens using paclitaxel showed a high success rate of 95–100% CIA prevention, while regimens using docetaxel generally showed a lower success rate of 43–96% alopecia prevention [62,63,64,65,67]. For scalp cooling to prevent CIA, the cap must be properly molded to the scalp to achieve the desired low temperature. A case report documented a woman treated with docetaxel who observed a 10 cm oval hair loss area after the first docetaxel cycle due to an improperly molded cap, and hair regrowth was noted after modifying the cap [68]. Scalp cooling administered by healthcare professionals remains the only FDA-cleared measure to prevent CIA by docetaxel. There have been concerns regarding scalp cooling increasing the risk of scalp metastasis. However, thus far, no statistically significant differences in the rates for scalp metastasis have been observed between scalp cooling and controls. A meta-analysis reported an incidence of 0.67% scalp metastasis with scalp cooling and 0.4% without scalp cooling (*p* = 0.43) [69]. Despite 77.5% of patients experiencing adverse effects, such as headache, dizziness, and scalp or neck pain [63], 70.2% of the patients surveyed were satisfied with using the scalp cooling systems [63]. Whereas some insurance plans reimburse the partial costs of FDA-cleared cooling systems, other plans do not. Therefore, the cost of these scalp cooling systems may be an issue that influences access. Self-administered scalp cooling caps also became available in recent years and offer lower costs and portability, but the outcome cannot be uniformly controlled due to user variability [14].

### Miscellaneous Novel Options

Other treatment options have been explored to address the need for better CIA treatment in the clinic (Figure 2). Cyclin-dependent kinases 4/6 (CDK4/6) inhibitors, such as palbociclib, are a novel orally administered preventative treatment for CIA due to their ability to pharmacologically induce cell cycle arrest in HFs [35,70,71,72,73,74]. Because taxanes initiate tumor cell death by directly interfering with the cell cycle, the ability to arrest the HF cell cycle makes the HFs less susceptible to taxane-induced damage in CIA [35,66,75,76,77,78,79,80]. A 2019 study found that administration of palbociclib led to a reversible arrest of proliferating hair matrix keratinocytes at the G1 phase, limiting the cytotoxic effects of paclitaxel on *ex vivo* human HFs and conveying the synergistic anticancer effect of CDK4/6 inhibitors with taxanes [35,77,79]. It is likely that CDK4/6 inhibitors could also limit docetaxel alopecia toxicity, as HFs respond similarly to docetaxel and paclitaxel [35]. In a study of patients treated with 60–75 mg/m^2^ docetaxel and oral 200 mg ribociclib (CDK4/6 inhibitor), 15 out of 43 (35%) patients developed alopecia, lower than the anticipated prevalence for docetaxel alone at this dose [77]. A phase Ib/II trial is currently investigating the efficacy of abemaciclib and paclitaxel combination in CDK4/6 pathway-activated tumors (NCT04594005). As CDK4/6 inhibitors are FDA-approved for metastatic breast cancer, the high cost can be covered to an extent by some insurance plans and/or with possible financial help from assistance programs [81].

In addition to the above approaches, other methods have also been investigated to prevent CIA. Recombinant keratinocyte growth factor, also called fibroblast growth factor-7 (KGF or FGF-7), has shown a dose-dependent cytoprotective effect against CIA with cytosine arabinoside chemotherapy in mice, reducing alopecia by 50% [82]. A clinical trial of KGF-hair serum for the prevention of CIA (NCT04554732) was completed in 2022 but the results are not yet published.

Low-intensity ultrasound has been tested as a preventative in cell culture and *ex vivo* human scalp HF organ culture treated with paclitaxel. The ultrasound eliminated acute cytotoxicity by disrupting the paclitaxel-induced rigid microtubules [83,84]. Because docetaxel has a mechanism of action similar to that of paclitaxel, low-intensity ultrasound may also protect against docetaxel-induced alopecia.

Additional agents that have shown efficacy for other chemotherapy regimens, including topical calcitriol (vitamin D), spironolactone, and topical vasoconstrictors, should also be explored for docetaxel treatment [14,34,58].

## 6. Research Models of CIA

Key Points:

- *Ex vivo* and *in vivo* models of CIA have been developed to get a better understanding of the underlying mechanisms of CIA and pCIA.
- A limitation of the murine model of CIA is the short anagen duration compared to human scalp HF, making it difficult to recapitulate human HF damage/recovery upon multi-course chemotherapy in the clinic.

Given the urgent need to understand the underlying mechanisms of CIA and pCIA, as well as test preventative or therapeutic approaches, various *ex vivo* and *in vivo* models of CIA have been developed. One well-established CIA research model is the *ex vivo* human scalp HF organ culture model, which allows researchers to directly study the impact of chemotherapeutic agents on human scalp HFs [85,86]. Human HFs are harvested from human scalp skin specimens, usually from patients undergoing plastic surgery (e.g. facelift) or hair transplantation, with ethical approval [85,86]. The anagen HFs are isolated and maintained in culture, where they can be manipulated with chemotherapeutic agents to study damage mechanisms to different cell types and test preventative methods [85,86]. Such studies have revealed mitotic defects and apoptosis in both the transit-amplifying hair matrix keratinocytes and Keratin 15-positive epithelial stem cells, implicating that direct damage to stem/progenitor cells could be the cause for the severity and permanence of CIA induced by paclitaxel and docetaxel [35]. This system has also been used to test a variety of agents to prevent chemotherapy damage to the HFs [83,84].

Another well-established research model of CIA is the neonatal rat or mouse model. This *in vivo* model takes advantage of the fact that during postnatal HF morphogenesis, the matrix cells undergo tremendous proliferation, making them susceptible to antineoplastic chemotherapy agents in a similar way to human scalp anagen HFs [50,87,88,89,90,91,92]. In addition, to better mimic the human scalp anagen HFs that have already undergone several hair cycles before they encounter chemotherapy, adult rat and mouse models have also been developed [93,94,95,96,97]. Widely used is a mouse model with depilation-induced anagen, in which the dorsal skin of 7-week-old mice is depilated with a wax/rosin mixture when the HFs are in telogen [95,96,97]. In 9 days, the depilated HFs enter anagen VI, mimicking the maximal susceptibility of human scalp HFs to antineoplastic agents, when the vast majority of scalp HFs are of anagen VI. Compared with spontaneous anagen development in a wave-like pattern, depilation-induced anagen is fully synchronized over the entire area of depilation [95].

A limitation of the murine model of CIA is the short anagen duration compared to human scalp HF, making it difficult to recapitulate human HF damage upon multi-course chemotherapy in the clinic when HFs are already in recovery at the time of the next treatment [98]. This limitation can be circumvented by using xenografted human scalp onto immunocompromised mice [60,99,100,101,102,103]. By grafting human scalp onto SCID/beige mice, we have observed human hair growth for more than 12 months after xenotransplantation. These mice can then be treated with chemotherapeutic agents and any hair loss or damage to the xenografted human HFs can be studied, as well as the effects of preventative or therapeutic agents. A limitation of the humanized mouse model is the lack of a fully functional immune system.

## 7. Conclusions

Docetaxel is a widely prescribed chemotherapeutic agent for the treatment of various types of cancers. One of its most common adverse effects is hair loss (presenting as CIA and pCIA), which most commonly affects women receiving docetaxel as monotherapy or combined therapy for breast cancer, which negatively impacts patients’ quality of life. To rectify the negative impacts of CIA and pCIA on patients, a variety of treatment modalities and preventative measures have been explored. Among these treatments are topical and oral minoxidil, photobiomodulation, and platelet-rich plasma injections, each showing variable degrees of success. The current preventative measures include FDA-cleared scalp cooling systems. Cyclin-dependent kinases 4/6 inhibitors and keratinocyte growth factor have also shown promise in early trials. Interdisciplinary collaboration between oncologists, dermatologists, and researchers will facilitate the effective prevention and/or treatment of CIA and pCIA. Work towards understanding the pathogenesis of CIA and pCIA is of utmost importance for future success.
